# Serum Albumin Before CRRT Was Associated With the 28- and 90-Day Mortality of Critically Ill Patients With Acute Kidney Injury and Treated With Continuous Renal Replacement Therapy

**DOI:** 10.3389/fnut.2021.717918

**Published:** 2021-08-25

**Authors:** Junhua Lv, Hai Wang, Baoni Sun, Yanxia Gao, Zhenglinag Zhang, Honghong Pei

**Affiliations:** ^1^Emergency Department, The Second Affiliated Hospital of Xi'an Jiaotong University, Xi'an, China; ^2^Department of Hepatobiliary Surgery, The First Affiliated Hospital of Xi'an Jiaotong University, Xi'an, China

**Keywords:** serum albumin, acute kidney infusion, 28- and 90-day mortality, critically ill patients, continuous renal replacement therapy

## Abstract

**Introduction:** Although low serum albumin (ALB) may worsen acute kidney injury (AKI), additional study is needed to establish the connection between ALB and the prognosis of critically ill patients with AKI and treated with continuous renal replacement therapy (CRRT).

**Methods:**A secondary analysis of a bi-center, retrospective, and observational study, such as critically ill patients with AKI and treated with CRRT from January 2009 to September 2016. The univariate analysis, multi-factor regression analysis, sensitivity analysis, and curve-fitting analysis were applied to explore the association of ALB with the 28 and 90 days mortality of critically ill patients with AKI and treated with CRRT, and the removal efficiency of serum phosphorus.

**Results:** From January 2009 to September 2016, 1,132 cases with AKI and treated with CRRT met the inclusion criteria and enrolled in this study. We found that the higher ALB before CRRT, the lower the 28- and 90-day mortality of patients with AKI and treated with CRRT, the higher removal efficiency of serum phosphorus, the adjusted hazard ratio (HR) value for 28-day mortality in the four models were separately 0.92 (0.90, 0.95), 0.91 (0.89, 0.94), 0.92 (0.89, 0.95), and 0.92 (0.89, 0.95); the adjusted HR value for 90 day mortality in the four models were 0.91 (0.89, 0.94), 0.92 (0.89, 0.95), 0.92 (0.89, 0.95), and 0.92 (0.89, 0.96); the adjusted OR value for the removal efficiency of serum phosphorus in the four models were separately −0.04 (−0.07, −0.01), −0.05 (−0.08, −0.01), −0.04 (−0.08, −0.01), and −0.04 (−0.08, −0.01). The sensitivity analysis and curve-fitting analysis also showed that ALB before CRRT was correlated with the 28 and 90 days mortality of critically ill patients with AKI and treated with CRRT and the removal efficiency of serum phosphorus.

**Conclusion:** The higher the serum ALB before CRRT, the lower the mortality of critically ill patients with AKI and treated with CRRT, and the higher the clearance efficiency of serum phosphorus.

## Introduction

Acute kidney injury (AKI) is a frequent complication of critically ill patients. Approximately 30–50% of critically ill patients develop AKI, and the mortality for individuals with AKI may reach 50% (1). Approximately 40% of the AKI patients may advance to life-threatening renal dysfunction (1, 2), such as hyperkalemia, severe acidosis, severe azotemia, oliguria, or continuous anuria, and will need renal replacement treatment, among which continuous renal replacement therapy (CRRT) is the most commonly used renal replacement therapy in the intensive care unit (ICU) (1). The efficiency of CRRT clearance is closely related to the prognosis of patients (3). According to various research studies, the serum phosphorus clearance of CRRT is linked to the serum creatinine and urea nitrogen clearance (4–6). Simultaneously, serum phosphorus clearance of CRRT is linked to the prognosis of critically ill patients (7, 8). ALB is one of the most critical proteins in the human plasma because it may maintain plasma colloid osmotic pressure, engage in material transport in blood circulation, and facilitate communication among the intracellular fluid, extracellular fluid, and tissue fluid (9). Hypoalbuminemia is widespread in patients with critical illnesses, and it is widely recognized as being linked to patient deterioration and higher death (10, 11). The incidence of hypoproteinemia in hospitalized patients is ~21% (12), but its incidence in acute illness is more than 50% (13).

In addition, ALB is closely related to the occurrence and progression of AKI. According to David R Williamson's study, ALB administration was linked with a dose-dependent risk of AKI associated with colloids after heart surgery (14). Low serum ALB was shown to be an independent risk factor for AKI in the meta-analysis of Michael Joannidis, which comprised 43 retrospective observational cohort studies that include 68,000 patients (15).

However, fewer studies have been conducted to investigate the relationship between serum ALB and the prognosis of critically ill patients with AKI and treated with CRRT, and it is unclear whether serum ALB affects the clearance efficiency of serum phosphorus. Therefore, this research assumes that the higher the serum ALB before CRRT, the lower the mortality of severe AKI patients and the higher the serum phosphorus clearance efficiency.

## Methods

### Study Design

A secondary analysis of a bi-center, retrospective, and observational study including critically ill patients with acute kidney injury (AKI) and treated with CRRT from January 2009 to September 2016 at Yonsei University Health System Severance Hospital and National Health Insurance Service Medical Center Ilsan hospital, Republic of Korea.

### Objective

The study aimed to explore the relationship of ALB with the 28- and 90-day mortality of patients with AKI and treated with CRRT and with the removal efficiency of serum phosphorus.

### Ethics Approval and Consent to Participate

New ethics permission and consent to participate were not applicable since the original author had received ethical approval while performing this research, and our study was a retrospective analysis of data reuse.

### Data Source

The data used in this study were shared by Seung Hyeok Han, which were stored in the Dryad database (https://datadryad.org//resource/doi:10.5061/dryad.6v0j9) (7). The database is a public data repository, which contains data uploaded by the authors to make their research data discoverable, freely reusable, and citable.

### Inclusion Criteria

(1) Patients were complicated with AKI and treated with CRRT in the intensive care unit (ICU); (2) the stage of AKI was two or more according to the acute kidney injury network (AKIN) criteria, in which serum creatinine and urine outputs were taken into account.

### Exclusion Criteria

Patients with the following situation were excluded in this study: (1) age <18 years; (2) pregnant or lactating women; (3) with postrenal obstruction; (4) with Stage 5 chronic kidney disease (CKD), kidney transplantation, dialysis, or CRRT; (5) the value of ALB was missing or outliers.

### Participants

**Figure 1:**
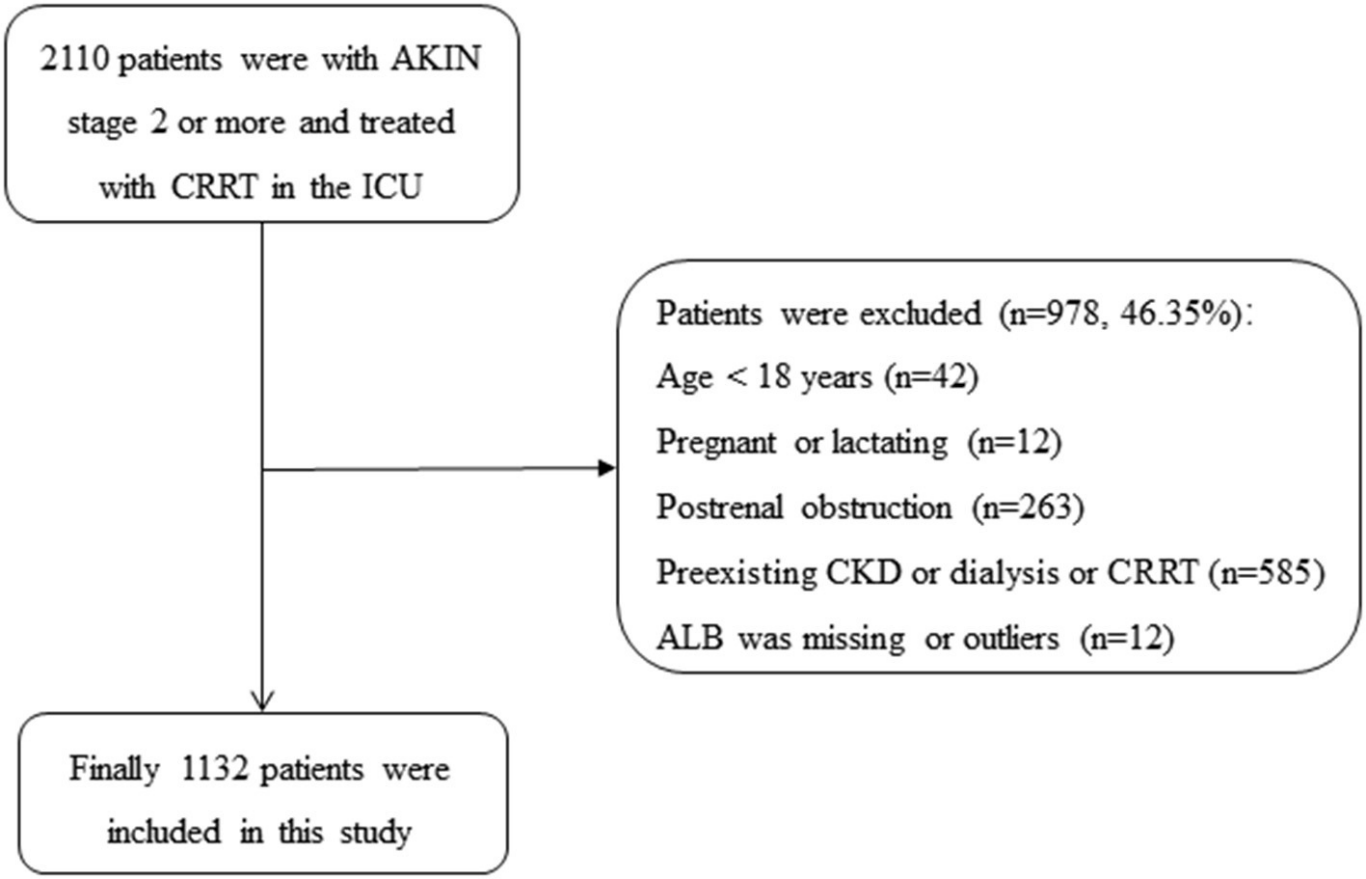
Flowchart of patient selection. AKIN, acute kidney injury network; ALB, albumin; CKD, chronic kidney diseases; CRRT, continuous renal replacement therapy; ICU, intensive care unit.

From January 2009 to September 2016, 2,110 patients were presented with AKIN stage 2 or more and treated with CRRT, of which 978 patients were excluded in the ICU at Yonsei University Health System Severance Hospital and National Health Insurance Service Medical Center Ilsan hospital: (1) age <18 years (*n* = 42); (2) pregnant or lactating women (*n* = 12); (3) with postrenal obstruction (*n* = 263); (4) with the history of stage 5 CKD, kidney transplantation, dialysis, or CRRT (*n* = 585); (5) the value of ALB was missing (*n* = 9) or outliers (*n* = 3), the ALB of two patients were 0 g/L, and the another was 5.9 g/L). Finally, a total of 1,132 patients with AKI and treated with CRRT were included in the current study (as shown in Figure 1).

### Grouping

According to ALB before CRRT, patients were divided into three groups: ALB <25 g/L (*n* = 436), 25 g/L ≤ ALB <30 g/L (*n* = 401), and 30 g/L ≤ ALB.

### The Outcome Indicators

(1) 28 and 90 days mortality; (2) the removal efficiency of serum phosphorus, delta phosphate = phosphate (24 h)-phosphate (0 h).

### Collection of Clinical and Biochemical Data

The following variables were included in the current study, such as age, sex, body mass index (BMI), mean arterial pressure (MAP), complications (myocardial infarction, congestive heart failure, cerebrovascular disease, peripheral vascular disease, dementia, diabetes mellitus, hypertension, and chronic obstructive pulmonary disease), Charlson comorbidity index (CCI) score, biochemical laboratory tests, e.g., K^+^, HCO3^−^, phosphate (0 h), phosphate (24 h), delta phosphate, white blood cell (WBC), hemoglobin (HB), blood urea nitrogen (BUN), creatinine (Cr) and C-reactive protein (CRP), and sequential organ failure assessment (SOFA) score. Age, gender, BMI, and CCI scores were collected at the time of admission; and the other variables were collected at 0 h of CRRT.

### CRRT Protocol

The nephrologist assessed whether the patients should be treated with CRRT based on the development of AKI in the ICU patients. The CRRT machines were the multiFiltrate (Fresenius Medical Care, Bad Homburg, Germany) or the Prismaflex (Baxter International Inc. Lundia AB, Sweden). The applied dialyzers had a surface area of 1.0–1.4 m^2^ with a sieving coefficient for albumin and ß2-microglobulin of 0.001 and 0.58–0.65, respectively. The parameters of CRRT were the following: (1) model: Continuous venovenous hemofiltration (CVVH) through the internal jugular, subclavian, or femoral vein; (2) blood flow: the start was at 100 ml/min and up to 150 ml/min; (3) The total dialysis and replacement dose were targeted to deliver ≥ 35 ml/kg/h in all the patients.

### Statistical Analysis

(1) Statistical description: Mean ± SD (x ± s) was used for the continuous variables of baseline data in the groups, and counts numerical values and percentages were shown in the data. The data were compared using the *t*-test, if continuous data had a normal distribution and homogeneity of variance. Mann–Whitney's *U*-test was performed if the continuous data did not meet the normal distribution or homogeneity of the variance. For categorical data, the χ^2^-test was utilized. (2) Analyze the relationship between ALB and 28- and 90-day mortality of patients, and delta phosphate by univariate and multivariate analysis. A multivariate Cox regression was performed for 28- and 90-day mortality, and multivariate logistic regression was performed for delta phosphate. (3) To further understand the relationship between ALB and 28- and 90-day mortality of patients, subgroup analyses were performed on the age, MAP, congestive heart failure, hypertension, AKIN stage, mechanical ventilation, SOFA score, AKI causes, and CRRT causes. (4) The selection of adjustment variables were by the following: if the confounders influenced the effective estimate of ALB by more than 10% and identified with the literature, we would adjust it. (5) Curve fitting analysis was used to investigate the connection among ALB, 28- and 90-day mortality, and delta phosphate. All statistical analyses were carried out by EmpowerStats 2.0 (Copyright 2009 X&Y Solutions, Inc.) and R software (version 3.4.3). The value of *P* < 0.05 was statistically significant.

## Results

### Baseline Characteristics

In this research, 1,132 patients with AKI and treated with CRRT were included. The ages of three groups included were as follows: ALB <25 g/L (*n* = 436), 25 g/L ≤ ALB <30 g/L (*n* = 401), and 30 g/L ≤ ALB were 63.31 ± 14.21, 64.27 ± 14.09, and 61.82 ± 14.89 years, respectively, with no significant difference (*P* = 0.091). The male-to-female ratio among the three groups was 280/156 (1.79), 241/160 (1.51), and 178/117 (1.52), with no statistical difference (*P* = 0.399). The BMI among the three groups was 23.52 ± 4.72, 23.75 ± 4.49, and 24.24 ± 4.48 kg/m^2^, respectively, with statistical significance (*P* = 0.034). The 28-day mortality among the three groups was 307 (70.41%), 257 (64.09%), and 141 (47.80%), respectively, with statistical significance (*P* < 0.001). The 90-day mortality among the three groups was 350 (80.28%), 294 (73.32%), and 171 (57.97%), respectively, with statistical significance (*P* < 0.001). There was a significant difference between myocardial infarction, congestive heart failure, and dementia (*P* < 0.05). There was a significant difference in HCO^3−^, phosphate (24 h), delta phosphate, HB, CRP, WBC, SOFA score, AKI cause, and CRRT cause across the three groups (*P* < 0.05). There was no significant difference in the other variables between the groups (*P* > 0.05) (as shown in Table 1).

**Table 1:** The clinical characteristics of patients.

**Variables**	**Alb <25 (*n* = 436)**	**25 ≤ Alb <30 (*n* = 401)**	**30 ≤ Alb (*n* = 295)**	***P-*value**
Age, year	63.31 ± 14.21	64.27 ± 14.09	61.82 ± 14.89	0.091
Sex (M/F)	280/156	241/160	178/117	0.399
BMI, kg/m^2^	23.52 ± 4.72	23.75 ± 4.49	24.24 ± 4.48	0.034
Myocardial infarction, *n* (%)	31 (7.11%)	39 (9.73%)	39 (13.22%)	0.023
Congestive heart failure, *n* (%)	54 (12.39%)	68 (16.96%)	63 (21.36%)	0.005
Cerebrovascular disease, *n* (%)	47 (10.83%)	44 (11.03%)	22 (7.46%)	0.233
Peripheral vascular disease, *n* (%)	19 (4.36%)	16 (3.99%)	10 (3.39%)	0.806
Dementia, *n* (%)	21 (4.82%)	17 (4.24%)	4 (1.36%)	0.041
Diabetes mellitus, *n* (%)	146 (33.49%)	145 (36.25%)	104 (35.25%)	0.698
Hypertension, *n* (%)	241 (55.28%)	211 (52.62%)	142 (48.14%)	0.165
COPD, *n* (%)	29 (6.65%)	29 (7.23%)	22 (7.46%)	0.905
CCI score	3.17 ± 2.40	3.19 ± 2.05	3.09 ± 2.25	0.418
K^+^, mmol/L	4.74 ± 1.11	4.58 ± 1.05	4.81 ± 1.13	0.016
HCO3^−^, mmol/L	15.76 ± 5.41	17.72 ± 5.82	17.46 ± 5.86	<0.001
Phosphate (0 h), mg/dL	5.79 ± 2.57	5.49 ± 2.05	6.03 ± 2.58	0.061
Phosphate (24 h), mg/dL	4.85 ± 2.56	4.24 ± 1.83	4.64 ± 2.52	0.025
Delta phosphate	−0.96 ± 2.40	−1.23 ± 1.81	−1.41 ± 2.62	0.043
MAP, mmHg	76.43 ± 14.51	78.13 ± 14.33	77.30 ± 14.70	0.533
Hb, g/dL	9.28 ± 2.12	9.66 ± 2.09	10.08 ± 2.45	<0.001
BUN, mg/dL	57.78 ± 31.84	56.18 ± 27.68	52.52 ± 29.91	0.020
Cr, mg/dL	2.70 ± 1.62	2.67 ± 1.39	2.84 ± 1.88	0.794
CRP, mg/dL	121.51 ± 108.85	120.20 ± 116.13	82.83 ± 91.19	<0.001
WBC, 10^9^/L	13.76 ± 12.768	16.04 ± 14.82	13.75 ± 8.74	0.038
SOFA score	12.33 ± 3.38	12.41 ± 3.40	11.40 ± 3.83	<0.001
2 h urine output before CRRT, ml	65.13 ± 99.67	72.94 ± 99.04	79.40 ± 111.15	0.087
AKI cause				<0.001
Sepsis, *n* (%)	324 (74.31%)	279 (69.58%)	189 (64.07%)	
Nephrotoxin, *n* (%)	14 (3.21%)	14 (3.49%)	8 (2.71%)	
Ischemia, *n* (%)	46 (10.55%)	31 (7.73%)	20 (6.78%)	
Surgery, *n* (%)	24 (5.50%)	37 (9.23%)	31 (10.51%)	
Others, *n* (%)	28 (6.42%)	40 (9.98%)	47 (15.93%)	
AKIN stages				0.866
2, *n* (%)	113 (25.92%)	102 (25.44%)	81 (27.46%)	
3, *n* (%)	323 (74.08%)	299 (74.56%)	214 (72.54%)	
Mechanical ventilation, *n* (%)	361 (82.80%)	318 (79.30%)	212 (72.11%)	0.002
CRRT cause				0.190
Volume overload, *n* (%)	48 (11.01%)	55 (13.72%)	55 (18.64%)	
Metabolic acidosis, *n* (%)	94 (21.56%)	86 (21.45%)	60 (20.34%)	
Hyperkalemia, *n* (%)	25 (5.73%)	14 (3.49%)	18 (6.10%)	
Uremia, *n* (%)	44 (10.09%)	44 (10.97%)	26 (8.81%)	
Oliguria, *n* (%)	110 (25.23%)	109 (27.18%)	72 (24.41%)	
Other, *n* (%)	115 (26.38%)	93 (23.19%)	64 (21.69%)	
CRRT does, ml/kg	36.87 ± 4.83	36.66 ± 4.99	36.27 ± 4.53	0.317
28-day death, *n* (%)	307 (70.41%)	257 (64.09%)	141 (47.80%)	<0.001
90-day death, *n* (%)	350 (80.28%)	294 (73.32%)	171 (57.97%)	<0.001

### ALB Was Associated With the 28- and 90-day Mortality of Patients With AKI and Treated With CRRT

When ALB was used as a continuous variable, the higher the serum ALB before CRRT, the lower the mortality of critically ill patients with AKI and treated with CRRT. The adjusted hazard ratio (HR) value for 28-day mortality in the four models were separately 0.92 (0.90, 0.95), 0.91 (0.89, 0.94), 0.92 (0.89, 0.95), and 0.92 (0.89, 0.95). The adjusted HR value for 90-day mortality in the four models were 0.91 (0.89, 0.94), 0.92 (0.89, 0.95), 0.92 (0.89, 0.95), and 0.92 (0.89, 0.96). When ALB was used as a classification variable and ALB <25 g/L as a reference, it was discovered that when 25 g/L ≤ ALB <30 g/L and 30 g/L ≤ ALB, the 28- and 90-day mortality of patients with AKI and treated with CRRT were substantially decreased (as shown in Table 2).

**Table 2:** Multivariate logistic regression analysis for 28- and 90-day mortality.

**Exposure**	**28-day mortality (Adjusted HR 95%CI)**	***P*-value**	**90-day mortality (Adjusted HR 95%CI)**	***P*-value**
**Model 1**
Alb, g/L	0.92 (0.90, 0.95)	<0.001	0.91 (0.89, 0.94)	<0.001
Alb, g/L
< 25	1.00 (Reference)		1.00 (Reference)	
25 ≤ and <30	0.71 (0.53, 0.96)	0.027	0.62 (0.45, 0.87)	0.006
30 ≤	0.37 (0.27, 0.51)	<0.001	0.32 (0.23, 0.45)	<0.001
**Model 2**
Alb, g/L	0.91 (0.89, 0.94)	<0.001	0.92 (0.89, 0.95)	<0.001
Alb, g/L
<25	1.00 (Reference)		1.00 (Reference)	
25 ≤ and <30	0.54 (0.36, 0.80)	0.002	0.53 (0.35, 0.81)	0.003
30 ≤	0.35 (0.23, 0.54)	<0.001	0.37 (0.24, 0.59)	<0.001
**Model 3**
Alb, g/L	0.92 (0.89, 0.95)	<0.001	0.92 (0.89, 0.95)	<0.001
Alb, g/L
<25	1.00 (Reference)		1.00 (Reference)	
25 ≤ and <30	0.57 (0.38, 0.87)	0.009	0.56 (0.36, 0.88)	0.011
30 ≤	0.35 (0.22, 0.56)	<0.001	0.39 (0.24, 0.63)	<0.001
**Model 4**
Alb, g/L	0.92 (0.89, 0.95)	<0.001	0.92 (0.89, 0.96)	<0.001
Alb, g/L
<25	1.00 (Reference)		1.00 (Reference)	
25 ≤ and <30	0.58 (0.37, 0.91)	0.017	0.59 (0.37, 0.94)	0.027
30 ≤	0.37 (0.22, 0.60)	<0.001	0.41 (0.24, 0.68)	<0.001

*Model 1: Adjusted for age; sex; body mass index (BMI); Charlson comorbidity index (CCI score). Model 2: Adjusted for model 1 plus C-reactive protein (CRP), white blood cell (WBC), hemoglobin (HB), phosphate (0 h), K^+^, HCO3-. Model 3: Adjusted for model 2 plus acute kidney injury (AKI) cause, continuous renal replacement therapy (CRRT) cause, (acute kidney injury network (AKIN) stages, CRRT does, 2 h urine output before CRRT initiation. Model 4: Adjusted for model 3 plus sequential organ failure assessment (SOFA) score*.

### The Sensitivity Analysis Was Used to Detect the Relationship Between the ALB and the 28- and 90-day Mortality of Patients With AKI and Treated With CRRT

Age, MAP, myocardial infarction, congestive heart failure, diabetes mellitus, hypertension, AKIN stage, mechanical ventilation, SOFA score, AKI causes, and CRRT causes were all subjected to sensitivity analysis. ALB was associated with 28-day mortality in the sensitivity analysis, except in individuals with myocardial infarction, AKIN stage 2, or CRRT cause (hyperkalemia, uremia, and oliguria). Additional sensitivity analysis for 90-day mortality revealed that ALB was linked with 90-day death in all the patients except those with myocardial infarction, congestive heart failure, AKIN stage 2, or CRRT cause (hyperkalemia, uremia, and oliguria; as shown in Table 3).

**Table 3:** The subgroup analysis of multivariate logistic regression analysis for 28- and 90-day mortality.

**Exposure**	**28-day mortality (Adjusted HR 95%CI)**	***P*-value**	**90-day mortality (Adjusted HR 95%CI)**	***P*-value**
**Age, year**
<65	0.95 (0.92, 0.98)	0.001	0.97 (0.94, 1.00)	0.031
65 ≤	0.96 (0.93, 0.98)	<0.001	0.94 (0.92, 0.97)	<0.001
**MAP, mmHg**
<65	0.91 (0.87, 0.95)	<0.001	0.94 (0.91, 0.98)	0.006
65 ≤	0.96 (0.94, 0.98)	<0.001	0.96 (0.94, 0.98)	<0.001
**Myocardial infarction**
Yes	1.05 (0.95, 1.17)	0.315	0.98 (0.88, 1.08)	0.638
No	0.96 (0.94, 0.98)	<0.001	0.96 (0.94, 0.98)	<0.001
**Congestive heart failure**
Yes	0.94 (0.90, 0.99)	0.018	0.961 (0.919, 1.004)	0.077
No	0.96 (0.94, 0.98)	<0.001	0.956 (0.938, 0.975)	<0.001
**Diabetes mellitus**
Yes	0.95 (0.92, 0.98)	<0.001	0.94 (0.91, 0.97)	<0.001
No	0.98 (0.96, 1.00)	0.111	0.97 (0.95, 0.99)	0.007
**Hypertension**
Yes	0.96 (0.94, 0.99)	0.005	0.95 (0.92, 0.97)	<0.001
No	0.95 (0.92, 0.97)	<0.001	0.96 (0.93, 0.98)	<0.001
**AKIN stage**
2	0.97 (0.93, 1.00)	0.081	0.97 (0.93, 1.00)	0.062
3	0.95 (0.93, 0.97)	<0.001	0.95 (0.93, 0.97)	<0.001
**Mechanical ventilation**
Yes	0.97 (0.95, 0.99)	0.001	0.96 (0.94, 0.98)	<0.001
No	0.95 (0.91, 1.00)	0.036	0.95 (0.91, 0.99)	0.027
**SOFA score**
<8	0.85 (0.74, 0.98)	0.026	0.86 (0.77, 0.96)	0.007
8 ≤ and <12	0.95 (0.92, 0.99)	0.006	0.95 (0.92, 0.98)	0.001
12 ≤	0.97 (0.94, 0.99)	0.011	0.97 (0.95, 0.99)	0.012
**AKI causes**
Sepsis	0.96 (0.94, 0.98)	<0.001	0.95 (0.93, 0.97)	<0.001
Non-sepsis	0.94 (0.90, 0.98)	0.004	0.95 (0.91, 0.98)	0.004
**CRRT causes**
Volume overload	0.89 (0.84, 0.94)	<0.001	0.91 (0.86, 0.95)	<0.001
Metabolic acidosis	0.95 (0.91, 0.99)	0.015	0.94 (0.90, 0.98)	0.002
Hyperkalemia	1.01 (0.86, 1.17)	0.935	1.03 (0.91, 1.18)	0.617
Uremia	1.02 (0.92, 1.13)	0.667	0.97 (0.88, 1.07)	0.549
Oliguria	1.00 (0.97, 1.05)	0.820	0.98 (0.95, 1.02)	0.292
Other	0.94 (0.91, 0.98)	0.005	0.96 (0.92, 0.99)	0.022

*Adjusted variables (without the subgroup analysis variables themselves): age; sex; body mass index (BMI); Charlson comorbidity index (CCI score), C-reactive protein (CRP), white blood cell (WBC), hemoglobin (HB), phosphate (0 h), K+, HCO3-, acute kidney injury (AKI) cause, continuous renal replacement therapy (CRRT) cause, acute kidney injury network (AKIN) stages, CRRT does, 2 h urine output before CRRT initiation, sequential organ failure assessment (SOFA) score*.

### ALB Was Associated With the Removal Efficiency of Phosphate

When ALB was used as a continuous variable, the higher the serum ALB before CRRT treatment, the higher the clearance efficiency of serum phosphorus. The adjusted OR values for delta phosphate in the four models were −0.04 (−0.07, −0.01), −0.05 (−0.08, −0.01), −0.04 (−0.08, −0.01), and −0.04 (−0.08, −0.01). When ALB was used as a classification variable, it was also found that the higher the ALB of patients, the higher the removal efficiency of serum phosphorus (as shown in Table 4).

**Table 4:** Multivariate logistic regression analysis for delta phosphate.

**Exposure**	**delta phosphate (Unadjusted OR 95%CI)**	***P*-value**	**delta phosphate (Adjusted OR 95%CI)**	***P-*value**
**Model 1**
Alb, g/L	−0.04 (−0.07, −0.01)	0.004	−0.04 (−0.07, −0.01)	0.003
Alb, g/L
<25	Reference		Reference	
25 ≤ and <30	−0.27 (−0.60, 0.06)	0.111	−0.24 (−0.58, 0.09)	0.153
30 ≤	−0.44 (−0.81, −0.07)	0.019	−0.48 (−0.85, −0.10)	0.012
**Model 2**
Alb, g/L	−0.04 (−0.07, −0.01)	0.004	−0.05 (−0.08, −0.01)	0.006
Alb, g/L
<25	Reference		Reference	
25 ≤ and <30	−0.27 (−0.60, 0.06)	0.111	−0.33 (−0.76, 0.10)	0.137
30 ≤	−0.44 (−0.81, −0.07)	0.019	−0.61 (−1.09, −0.13)	0.013
**Model 3**
Alb, g/L	−0.04 (−0.07, −0.01)	0.004	−0.04 (−0.08, −0.01)	0.010
Alb, g/L
<25	Reference		Reference	
25 ≤ and <30	−0.27 (−0.60, 0.06)	0.111	−0.24 (−0.68, 0.19)	0.271
30 ≤	−0.44 (−0.81, −0.07)	0.019	−0.60 (−1.08, −0.11)	0.016
**Model 4**
Alb, g/L	−0.04 (−0.07, −0.01)	0.004	−0.04 (−0.08, −0.01)	0.014
Alb, g/L
<25	Reference		Reference	
25 ≤ and <30	0.27 (−0.60, 0.06)	0.111	−0.23 (−0.67, 0.20)	0.293
30 ≤	−0.44 (−0.81, −0.07)	0.019	−0.58 (−1.06, −0.09)	0.020

*Model 1: Adjusted for age; sex; body mass index (BMI); Charlson comorbidity index (CCI score). Model 2: Adjusted for model 1 plus C-reactive protein (CRP), white blood cell (WBC), hemoglobin (HB), K+, HCO3-. Model 3: Adjusted for model 2 plus acute kidney injury (AKI) cause, continuous renal replacement therapy (CRRT) cause, acute kidney injury network (AKIN) stages, CRRT does, 2 h urine output before CRRT initiation. Model 4: Adjusted for model 3 plus sequential organ failure assessment (SO-0+FA) score*.

### The Relationship Among ALB and 28-, 90-day Mortality, and the Removal Efficiency of Serum Phosphorus Explored by Curve Fitting Analysis

**Figure 2:**
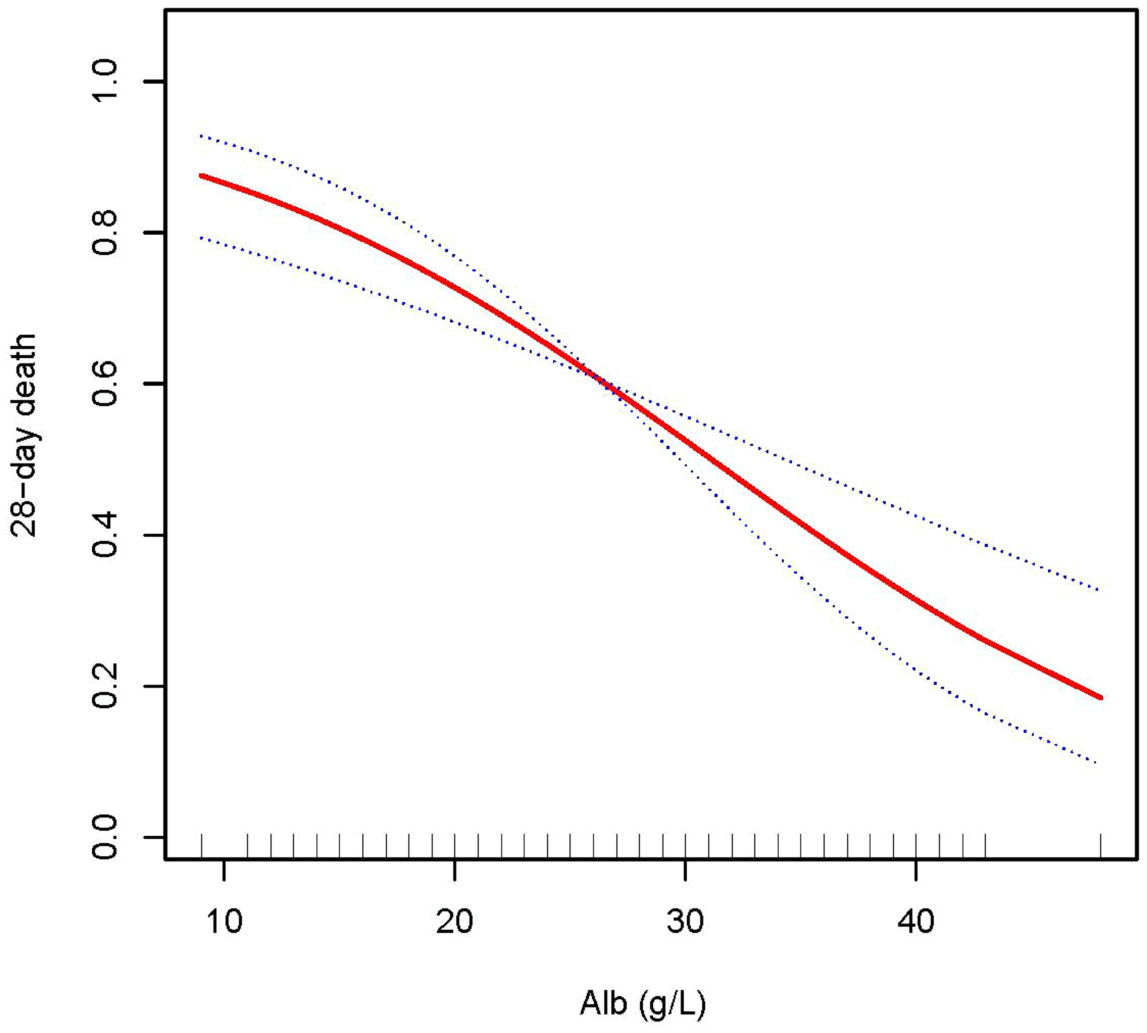
Adjusted smoothing function of ALB for 28-day mortality. After adjusting for age, gender, body mass index (BMI), Charlson comorbidity index (CCI score), C-reactive protein (CRP), white blood cell (WBC), hemoglobin (HB), phosphate (0 h), K^+^, HCO^3−^, acute kidney injury (AKI) cause, continuous renal replacement therapy (CRRT) cause, (acute kidney injury network (AKIN) stages, CRRT dose, 2 h urine output before CRRT initiation, and sequential organ failure assessment (SOFA) score, curve fitting analysis showed that the 28-day mortality rate decreased as the ALB increased.

**Figure 3:**
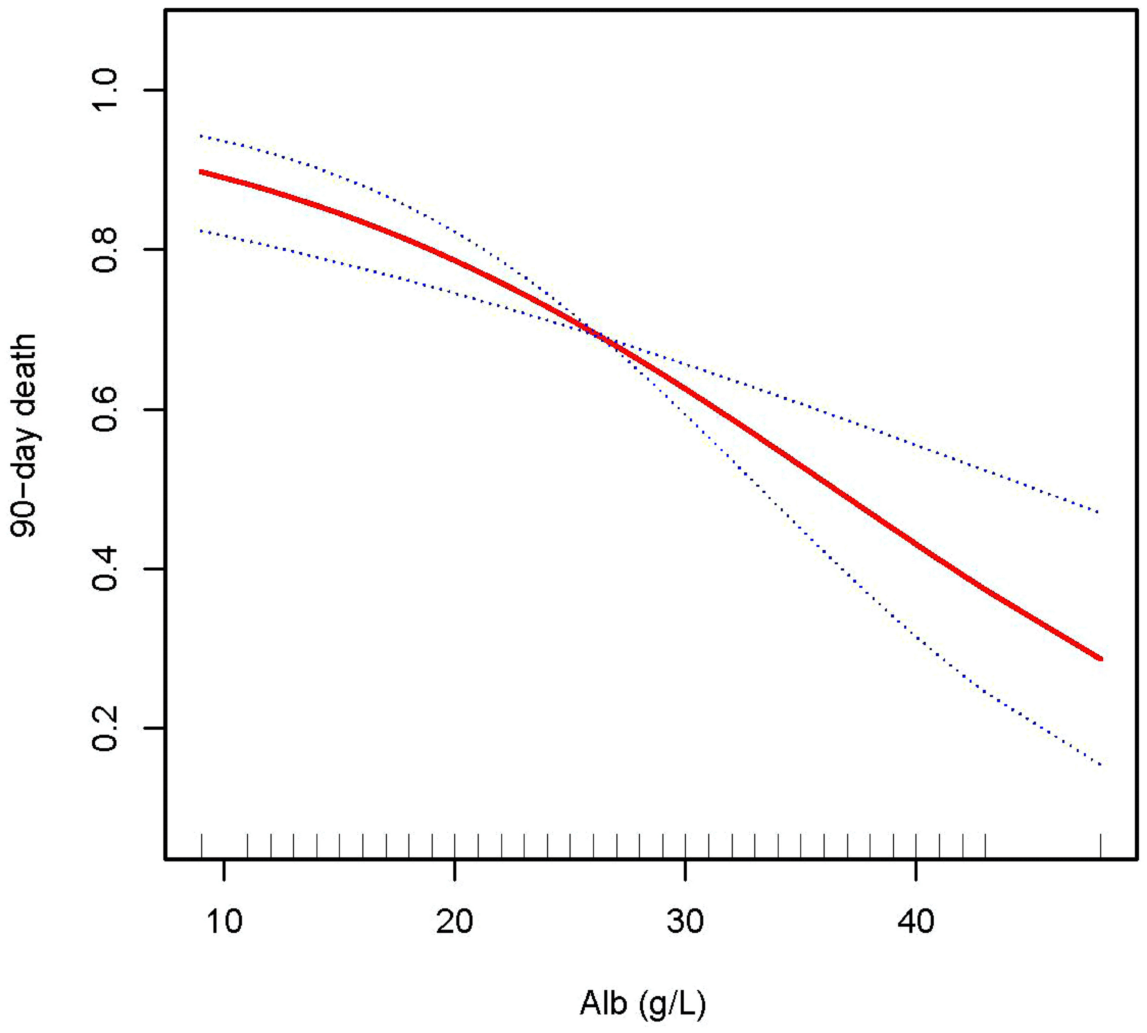
Adjusted smoothing function of ALB for 90-day mortality. After adjusting for age, gender, body mass index (BMI), Charlson comorbidity index (CCI score), C-reactive protein (CRP), white blood cell (WBC), hemoglobin (HB), phosphate (0h), K^+^, HCO^3−^, acute kidney injury (AKI) cause, continuous renal replacement therapy (CRRT) cause, (acute kidney injury network (AKIN) stages, CRRT dose, 2 h urine output before CRRT initiation, andsequential organ failure assessment (SOFA) score, curve fitting analysis showed that the 90-day mortality rate decreased as the ALB increased.

**Figure 4:**
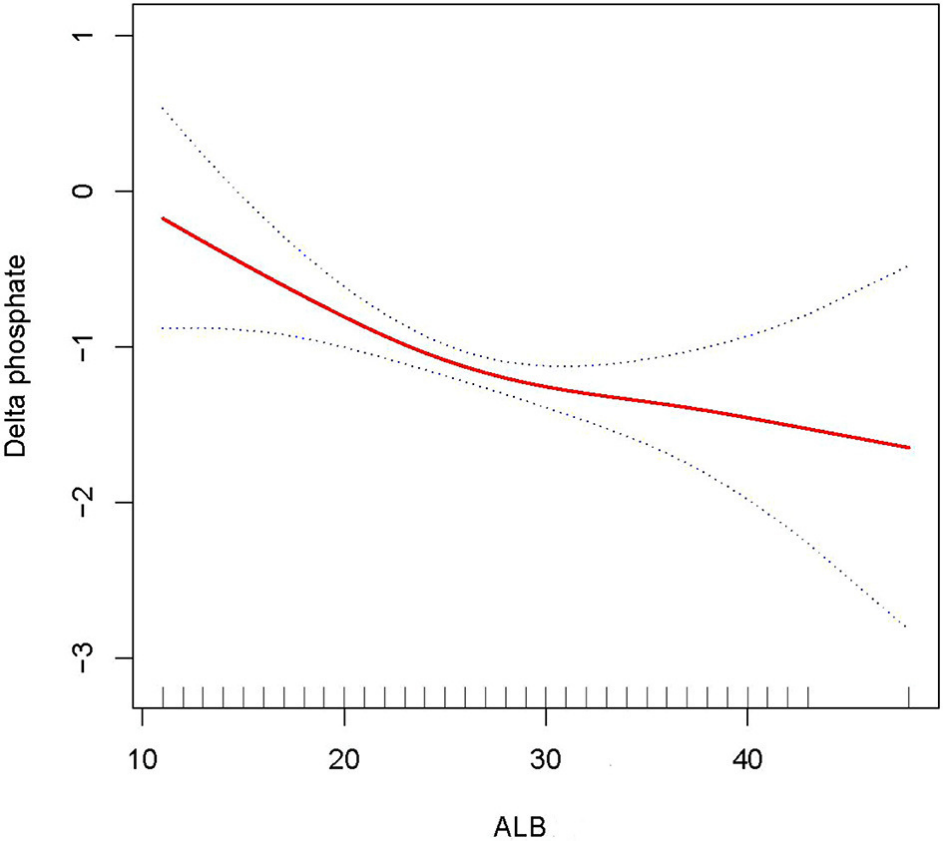
Adjusted smoothing function of ALB for delta phosphate. After adjusting for age, gender, body mass index (BMI), Charlson comorbidity index (CCI score), C-reactive protein (CRP), white blood cell (WBC), hemoglobin (HB), K^+^, HCO^3−^, acute kidney injury (AKI) cause, continuous renal replacement therapy (CRRT) cause, (acute kidney injury network (AKIN) stages, CRRT dose, 2 h urine output before CRRT initiation, and sequential organ failure assessment (SOFA) score, curve fitting analysis showed that delta phosphate increased as the ALB increased.

In this study, we discovered that the higher the ALB, the lower the 28- and 90-day mortality of patients with AKI treated with CRRT, and the higher the delta phosphate in the curve fitting analysis. The following variables were adjusted in curve fitting analysis: age, sex, BMI, CCI, CRP, WBC, HB, phosphate (0 h) (except for delta phosphate), k^+^, HCO^3−^, AKI cause, CRRT cause, AKIN stages, CRRT dose, 2 h urine output before CRRT, and SOFA score (as shown in Figures 2–4).

## Discussion

According to this study, the higher the ALB before CRRT, the lower the 28- and 90-day mortality of patients with AKI treated with CRRT. It was also discovered that the higher the ALB before CRRT, the greater the serum phosphorus clearance efficiency.

Hypoalbuminemia had been demonstrated in certain studies to exacerbate AKI and to deteriorate the prognosis of individuals with AKI. According to Michael Joannidis's study, hypoalbuminemia accelerated the progression of AKI, increased the need for CRRT therapy for AKI patients, and was a significant predictor of AKI incidence (OR: 2.96, 95% *CI*: 2.05–4.26) and mortality (OR: 2.47, 95% *CI*: 1.51–4.05) (16). A retrospective cohort study of 381 critically ill patients by Praveen Kolumam Parameswaran also found that serum hypoproteinemia was an independent risk factor for AKI (OR: 1.810, 95% *CI*: 1.102–2.992) in critically ill patients (17). It also promoted the progress of AKI to CKD (17). In addition, we constructed a prediction model about AKI, and they found that ALB was an independent predictor of 28-day mortality of AKI patients (18). The possible mechanisms are as follows: (1) Plasma colloid osmotic pressure was critical in controlling the exchange of water between the inside and outside of blood vessels and in maintaining blood volume, while albumin was the primary molecule responsible for sustaining plasma colloid osmotic pressure; (2) Hypoalbuminemia, when accompanied by blood volume reduction causes the liquid in blood vessels to leak out, further reducing the volume of blood vessels and aggravating renal perfusion, along with worsening acute kidney damage (7, 8, 18–20).

Continuous renal replacement therapy is often utilized in renal replacement therapy for critically ill patients, especially for those with hemodynamic instability (21). CRRT treatment for critically ill patients removes excess water and certain potentially toxic macromolecular compounds (22). Several studies had shown that insufficient CRRT treatment would result in adverse outcomes for patients. Simultaneously, it raised the risk of re-CRRT therapy, thus increasing the medical risk, treatment cost, and length of stay (23). According to studies, serum phosphorus clearance in CRRT was positively associated with and reasonably near to the serum creatinine and urea nitrogen clearance in CRRT (4–6). Meanwhile, investigations had indicated that phosphate (0 h), phosphate (24 h), and delta phosphate were all linked to a higher risk of death in critically ill patients with septic AKI undergoing CRRT (7, 8, 18, 19). This study found that the higher serum ALB, the more serum phosphorus decreased after CRRT. However, since there were no indications of creatinine or urea nitrogen following CRRT therapy in this research, the connection between albumin and the clearance efficiency of CRRT treatment was not yet established and needed to be further investigated and validated.

Hypotension often occurs during the early stages of CRRT. The primary cause of hypotension in the early stages of CRRT is insufficient blood vessel content (24). Serum ALB is required to maintain enough blood vessel content. The higher the serum ALB, the more abundant the blood vessel content, so the lower the risk of hypotension during CRRT. According to studies, hypotension 1 h after the start of CRRT increased the hospital mortality and was an independent predictor of hospital mortality (25). To summarize, it was considered that the higher serum ALB might enhance the prognosis of critically ill patients with AKI and treated with CRRT: (1) The higher serum ALB, the higher the clearance efficiency of serum phosphorus; (2) The higher the serum ALB, the more the blood vessel content, and the lower risk of hypotension during CRRT.

Age, MAP, myocardial infarction, congestive heart failure, diabetes mellitus, hypertension, AKIN stage, mechanical ventilation, SOFA score, AKI causes, and CRRT causes were all subjected to the sensitivity analysis. It was found that ALB was associated with the 28-day mortality, except for patients with myocardial infarction, AKIN stage 2, and CRRT cause (hyperkalemia, uremia, and oliguria). The potential explanation was that the risk of mortality from myocardial infarction was very high, masking the effect of serum ALB; several studies indicated that patients with AKIN stage 2 who had CRRT could not improve their prognosis (1, 26), which may be the reason why serum ALB did not affect the prognosis of patients with myocardial infarction and AKIN stage 2 undergoing CRRT in this study. Studies had also shown that hyperkalemia, oliguria, or anuria were not related to patient mortality, which was similar to the findings of this research (27, 28).

### Strength of the Study

(1) Through multivariate analysis, sensitivity analysis, and adjusting the potential confounding factors, this study got a more consistent conclusion: The higher the serum ALB, the better the prognosis of patients with AKI and treated with CRRT; (2) This study also made it clear that increasing the serum ALB might improve the clearance efficiency of serum phosphorus, and then improve the prognosis of critically ill patients with AKI and treated with CRRT.

### Limitations of the Study

(1) This research belongs to a bi-center, retrospective, and observational cohort study and lacked important data such as length of the sessions, how many sessions throughout the ICU stay, and methods for coagulation. This lead to a certain possible danger of bias in this research, so its conclusion needed to be verified by a prospective study. (2) The AKIN standard was used to diagnose acute renal injury in this research; however, Kidney Disease Improving Global Outcomes (KDIGO) may be a superior AKI diagnostic standard. (3) Due to the lack of data on creatinine and urea nitrogen after CRRT in this study, only the relationship between ALB and serum phosphorus clearance was obtained in this study, while the relationship between ALB and CRRT clearance efficiency was needed to be further studied. (4) Albumin will rely on the condition of extracellular hydration and blood volume, although hydration quantity, nutritional status of the patients, and exogenous protein supplements might influence albumin measurements, and lead to a certain potential risk of bias. (5) In addition, some patients lost to follow-up, which increased the possibility of bias in the results.

## Conclusion

The higher the serum ALB before CRRT, the lower the mortality of critically ill patients with AKI and treated with CRRT, and the higher the clearance efficiency of serum phosphorus.

## Data Availability Statement

The datasets presented in this study can be found in online repositories. The names of the repository/repositories and accession number(s) can be found at: (https://datadryad.org//resource/doi:10.5061/dryad.6v0j9).

## Ethics Statement

Agreement to participate in the study was not required because our review was a retrospective study of data reuse, and the patients' data was anonymous. Ethical approval was not provided for this study on human participants. The original author had obtained ethical approval when conducting his study. The ethics committee waived the requirement of written informed consent for participation in this study.

## Author Contributions

JL and HW participated in the research design, the revision of the manuscript, and data analysis. BS participated in the data analysis and writing of the paper. YG and ZZ participated in improving and revising the paper. HP provided substantial advice in designing the study and assisting in the division of labor, writing, and revising the paper. All authors contributed to the article and approved the submitted version.

## Conflict of Interest

The authors declare that the research was conducted in the absence of any commercial or financial relationships that could be construed as a potential conflict of interest.

## Publisher's Note

All claims expressed in this article are solely those of the authors and do not necessarily represent those of their affiliated organizations, or those of the publisher, the editors and the reviewers. Any product that may be evaluated in this article, or claim that may be made by its manufacturer, is not guaranteed or endorsed by the publisher.

## Abbreviations

CRRT: continuous renal replacement therapy
ALB: albumin
AKI: acute kidney injury
AKIN: acute kidney injury network
CKD: chronic kidney disease
ICU: intensive care unit
BMI: body mass index
MAP: mean arterial pressure
CCI score: Charlson comorbidity index
COPD: chronic obstructive pulmonary disease
WBC: white blood cell
HB: hemoglobin
BUN: blood urea nitrogen
Cr: creatinine
CRP: C-reactive protein
SOFA: sequential organ failure assessment
CVVH: continuous venovenous Hemofiltration.
